# Insufficient Sleep Syndrome: A Blind Spot in Our Vision of Healthy Sleep

**DOI:** 10.7759/cureus.30928

**Published:** 2022-10-31

**Authors:** Edward C Mader, Annie Cielo L Mader, Prachi Singh

**Affiliations:** 1 Department of Neurology, Louisiana State University Health Sciences Center, New Orleans, USA; 2 Science and Math Division, Delgado Community College, New Orleans, USA; 3 Sleep and Cardiometabolic Health Laboratories, Louisiana State University Pennington Biomedical Research Center, Baton Rouge, USA

**Keywords:** sleep, deprivation, restriction, insufficient, behavioral, public health, school, electronic devices, icsd, icd

## Abstract

Chronic sleep deficiency (CSD) poses a threat to physical health, mental well-being, and social functioning. The concept of behaviorally induced CSD has not changed much since it was first introduced four decades ago. Behaviorally induced CSD is currently referred to as insufficient sleep syndrome (ISS). In the latest edition of the International Classification of Sleep Disorders (ICSD-3, 2014), ISS is considered a disorder of central hypersomnolence with diagnostic codes ICD-9-CM 307.44 and ICD-10-CM F51.12. In this review, we will describe the biological importance of sleep, the ramifications of CSD on the individual and society, the nosological status and diagnostic features of ISS, and the apparent lack of attention to ISS in contemporary medical practice and public health programs. The last three decades have seen a global rise in voluntary sleep curtailment such that ISS may already be the leading cause of CSD, not only in adults but also in school-aged children and adolescents. Acknowledging ISS as a public health priority is a necessary first step in our response to the global threat of CSD and CSD-related health consequences. It is only by confronting ISS directly that we can hope to develop and implement effective educational and advocacy programs, along with more responsible public health policies and regulations.

## Introduction and background

Sleep is a physiologic state that is distinct from, but certainly not less important than, wakefulness. The fact that we must sleep to function effectively during wakefulness indicates that sleep is a biological necessity. Evidently, certain physiologic processes involved in restoring homeostasis, molecular biosynthesis, and building neural circuits operate more efficiently during sleep [1,2]. The biological functions of sleep can be divided into four categories (Table 1): (1) metabolic and functional recovery, (2) defense and response to injury, (3) neurodynamics and neuroplasticity, and (4) bioperiodicity and timing of biological processes [3]. These functional categories are not mutually exclusive. For example, sleep-induced neuroplasticity is also involved in acute functional recovery and response to injury. Moreover, the most neural activity involves neurodynamic and metabolic processes such that separating these two facets of physiology in order to categorize sleep function is purely arbitrary. Although the list of sleep functions in Table 1 is not exhaustive, the functions that are listed are adequate to convince us that it is not possible to survive and stay healthy without good sleep.

**Table 1:** The biological functions of sleep. Adapted with permission from Mader EC Jr and Mader AC [3].

The four main categories of sleep functions	
Metabolic and functional recovery	
	Energy balance: energy conservation by lowering metabolic rate and downscaling sensory and motor processes; restoration of energy stores
	Functional recovery: synthesis of proteins involved with neurotransmitter function, transport, membrane trafficking, lipid and myelin metabolism
	Waste removal: slowing metabolite buildup to enable cellular waste removal; increased glymphatic clearance
Defense and response to injury	
	Protective sleep behavior: sleeping` at the right time in a secure place increases survival chances
	Inflammatory and immune response: acute phase response to infection, inflammatory cytokine production (e.g., interleukins, TNF) and immuno-regulation
	Healing: promotes healing directly or indirectly via restorative, immune, and other functions
Neurodynamics and neuroplasticity	
	Learning and memory: strengthening of synapses and synaptic homeostasis to renormalize synapses in preparation for waking experience
	Developmental plasticity: rapid brain development and neurogenesis
	Neurodynamic homeostasis: for optimal neural signaling in wakefulness
Timing of biological processes	
	Programmed sleep behavior: reduces predation risk; enhances foraging success and reproductive capacity during wake periods
	Platform for synchronizing diverse processes: allows disparate biological processes to operate in harmony to achieve optimal health

Healthy sleep implies sufficient sleep quantity and good sleep quality. Sleep insufficiency occurs because of acute (total) sleep deprivation, short-term (1-3 days) acute (partial) sleep restriction, or chronic (repeated, usually >3 days) sleep restriction. Sleep deprivation or restriction increases sleep pressure, triggers a homeostatic response, and leads to subjective and objective sleepiness, reduced vigilance, and impaired cognitive and metabolic function [4,5]. Daily or near-daily sleep restriction over a period of weeks, months, or years will give rise to a state of chronic sleep deficiency (CSD). It remains unclear whether allostatic adaptation occurs during CSD in humans [6]. The number of hours per day and the number of days in which sleep must be curtailed to diagnose CSD in humans are also not well defined. At any rate, the cutoffs will most likely depend on age, health status, genetics, and other factors [5,7,8]. Clinically, CSD should be suspected if a person’s habitual daily sleep duration (usually the average of several weeks) is below the age-appropriate minimum daily sleep duration in accordance with the recommendations of the National Sleep Foundation (Table 2) [9]. One important caveat is that subjective sleepiness can be mild or absent in CSD [10,11]. Therefore, subjective sleepiness is a reliable indicator of acute sleep deprivation, but not of CSD.

**Table 2:** Sleep duration (in hours) recommended by the National Sleep Foundation. The recommendations define sleep time as (a) recommended, (b) may be appropriate for some individuals, or (c) not recommended. The recommendations are based on a rigorous and systematic review of a large number of studies in the world scientific literature. The range “may be appropriate” was added to acknowledge individual variability in sleep durations. Adapted with permission from Hirshkowitz M et al. [9].

Age group	Recommended	May be appropriate	Not recommended
Newborns (0-3 months)	14-17	11-13 or 18-19	<11 or >19
Infants (4-11 months)	12-15	10-11 or 16-18	<10 or >18
Toddlers (1-2 years)	11-14	9-10 or 15-16	<9 or >16
Preschoolers (3-5 years)	10-13	8-9 or 14	<8 or >14
Children (6-13 years)	9-11	7-8 or 12	<7 or >12
Teenagers (14-17 years)	8-10	7 or 11	<7 or >11
Young adults (18-25 years)	7-9	6 or 10-11	<6 or >11
Adults (26-64 years)	7-9	6 or 10	<6 or >10
Older adults (≥65 years)	7-8	5-6 or 9	<5 or >9

CSD can occur due to primary sleep disorders or to a wide range of psychiatric, neurological, or medical disorders. Of the many causes of CSD, the ones that garnered the most attention from healthcare providers and the public are sleep-related breathing disorders (SBD), especially obstructive sleep apnea (OSA), and chronic insomnia syndromes, notably insomnia due to inadequate sleep hygiene [12]. Paradoxically, the volitional and behavioral form of CSD, known as behaviorally induced CSD or insufficient sleep syndrome (ISS), is not getting the attention it deserves despite its pervasiveness in today’s society. In this article, we will review the consequences of CSD and the nosological status and diagnostic features of ISS. We will then explore the apparent lack of attention to ISS among patients and healthcare providers. Finally, we will describe public-oriented and clinic-based strategies for promoting health by directly confronting ISS.

## Review

Chronic sleep deficiency

Threats to the Person's Health and Social Functioning

The immediate threat of CSD to the individual is cognitive and behavioral in nature [4]. CSD decreases vigilance and disrupts other cognitive functions, leading to delayed reaction time, degraded motor performance, lower stress tolerance, anxiety, and impaired judgment [4,13,14]. These neurobehavioral effects of CSD increase the risk of accidents, physical injury, and social misbehavior, leading to problems in the workplace or school [15-18]. In humans, sleep loss has been linked to neuronal loss, changes in brain plasticity, morphometry, and network structure, and an increased risk of dementia [19-23]. Sleep deprivation is also known to increase the frequency of epileptiform discharges and clinical seizures in patients with epilepsy [24]. Animal studies demonstrated that prolonged sleep loss disrupts neurogenesis and hippocampal integrity leading to cognitive and mood disturbances [25]. These detrimental effects may be mediated by stress and glucocorticoids. During childhood and adolescence, CSD may result in impaired brain development and permanent loss of developmental potential [26-28].

CSD causes dysregulation of endocrine, metabolic, and inflammatory processes. There is a large body of epidemiological and experimental evidence linking CSD to increased food intake, obesity, insulin resistance, and hypertension leading to the development of diabetes and cardiovascular diseases [5,29-32]. Similar deleterious effects of CSD on dietary habits, glucose metabolism, and obesity have been observed in children and adolescents [33,34]. Sleep loss was found to increase the levels of inflammatory mediators and impair the immune response to infection and vaccines [35]. The pro-inflammatory response, heightened oxidative stress, blood-brain barrier disruption, and vascular endothelial cell dysfunction in CSD result in chronic low-grade inflammation and contribute to the pathogenesis of cardiometabolic disorders [36-38]. Other pathologies, such as the increased risk of osteoporosis and poor pregnancy outcomes, have also been linked to CSD [39-42]. Meta-analysis of prospective cohort studies revealed a link between short sleep duration and increased mortality [30,43,44]. A bidirectional relationship between CSD and psychiatric disorders has also been noted [45].

The behavioral and physiological manifestations of CSD may vary depending on the circadian phase and on the influence of non-circadian non-homeostatic factors, such as caffeine intake and exercise [3,46]. The clinical expression of CSD may also vary because of interindividual differences in sleep pressure adaptation and sleep loss recuperation [47]. Of particular importance is the bidirectional interaction between CSD and other diseases which adds another layer of complexity to any attempt at understanding the relationship between CSD and certain diseases [48].

Demographic Trends in Sleep Duration

The prevalence of CSD is roughly proportional to the prevalence of short sleep duration. What is considered abnormally “short sleep” depends on age but other factors, such as genetics, sleep quality, circadian pattern, general health, nutritional status, metabolic rate, and physical activity, may also have a role in shaping the body’s minimum sleep requirement. For healthy adults (age 18-64), <6 h of sleep per day is considered unhealthy by the National Sleep Foundation (Table 2) [9]. Most population-based studies examining the effects of sleep duration on health are cross-sectional surveys that employ subjective methods, such as self-reports, questionnaires, and time-use diaries. The majority of these studies found that short sleep duration is pervasive in many countries with a significant proportion of the population reporting <6 h of sleep per day regardless of gender [49-52]. Based on data from the 2016-2018 National Survey of Children’s Health, the CDC concluded that short sleep duration is prevalent among infants, children, and adolescents [53,54]. A recent longitudinal analysis of data from the US National Health Interview Survey between 2004 and 2017 showed a stable prevalence rate of short sleep duration between 2004 and 2012 and an increasing trend toward short sleep between 2013 and 2017 [55]. However, a global trend toward shorter sleep duration could not be established due to conflicting data from different countries [56]. In women, menopausal status contributes to sleep disturbances with the perimenopausal and menopausal periods showing the highest risk for CSD [57].

Economic status and occupation can predispose to short sleep duration. The odds of short sleep duration were increased in full-time workers, males, and those with high school diplomas or some college education [58]. Population-based studies of US workers found an increased prevalence of short sleep duration in those with long or extended work hours (>40 hours per week), those with rotating or shift work, and those with a high level of job-related stress [58,59]. The American Time Use Survey of 124,517 individuals showed that sleep time was frequently exchanged with paid work time and time spent commuting to and from work [60]. 

Ethnicity, culture, and psychosocial stressors have all been identified as determinants of sleep duration and quality [61]. In the US, non-Hispanic blacks, American Indians, Alaskan natives, and native Hawaiians, and Pacific Islanders are most likely to report insufficient nightly sleep [49]. A clustering of factors contribute to poor and insufficient sleep in individuals with low socioeconomic status, including working multiple jobs, food insecurity, lack of health insurance, and concerns related to neighborhood safety [62]. Cultural factors also influence sleep on a global scale. Later bedtimes and bed or room sharing with infants and other family members can adversely affect sleep duration and quality, especially in Asian countries where such practices are common [63]. 

Differences in perception and attitudes toward sleep may account for some of the demographic disparities in sleep health [62,64]. Attitudes toward sleep are often misguided because humans exhibit overconfidence in their abilities. While some individuals can function well in the face of short sleep duration, most people experience detrimental health and functional consequences if they do not get enough sleep. Urban dwellers frequently trade sleep for activities they perceive as more pressing, such as work or education, or as more gratifying, such as socializing or gaming. Sleep patterns and the perception of normal sleep are strongly influenced by sociocultural and environmental factors [60,62].

Threats to Public Health and Overall Economic Burden

The public health impact of insufficient sleep is well established. CSD predisposes to occupational or traffic-related injuries, lost productivity, impaired mood and social functioning, and increased healthcare utilization [16-18,65]. CSD can also have far-reaching impacts on public health by increasing the risk of chronic diseases, such as obesity, diabetes mellitus, cardiovascular disease, and perhaps cancer and other diseases characterized by immune dysregulation [5,29-32]. The prevalence of short sleep and the trend toward decreasing sleep duration in children and young adults are particularly concerning because of their long-term impact on public health [27,53,54,56]. The threat of CSD to public safety is more palpable in occupations related to transportation, law enforcement, and health care [62].

The economic burden of CSD is substantial. It has been estimated that approximately $680 billion is lost due to insufficient sleep across Canada, the United States, the United Kingdom, Germany, and Japan [66]. The aggregate total direct and indirect annual healthcare cost for insomnia alone has been estimated to be $100 billion in the US [67]. The cost of CSD in occupational settings has been evaluated as well. In a study of more than 4000 employees, Rosekind and colleagues showed that, compared to an employee who is well rested, an employee with insomnia or insufficient sleep can cost an employer twice as much in terms of annual productivity loss [68]. The loss of productivity is reflected in various areas of functioning, including time management and mental or physical job demands. Self-reported poor sleep quality has also been associated with higher healthcare costs [69]. The healthcare cost per person per year of individuals who reported difficulty sleeping always was on average $5,206 more than that of individuals who reported never having sleep problems [69].

Insufficient sleep syndrome

Nosological Status: Past and Present

The nosological evolution of ISS, insomnia, and inadequate sleep hygiene in the past 50 years is reflected in the periodic revisions of the International Classification of Diseases (ICD) [70], the Diagnostic and Statistical Manual of Mental Disorders (DSM) [71], and the International Classification of Sleep Disorders (ICSD) [12] (Table 3). Sleep disorders first appeared in the eighth ICD edition (ICD-8, 1965) under “Certain symptoms referable to the nervous system and special senses.” The second DSM edition (DSM-II, 1968) added “Disorders of sleep” under “VII. Special symptoms.” In 1979, the Association of Sleep Disorders Centers and the Association for the Psychophysiological Study published the first classification system dedicated to sleep disorders: the Diagnostic Classification of Sleep and Arousal Disorders (DCSAD, 1979) [72]. In 1990, the DCSAD was superseded by the first ICSD edition (ICSD-1, 1990) and its revision (ICSD-R, 1997). The ICSD was updated to its second edition (ICSD-2, 2005) and the ICSD-2 to the third edition (ICSD-3, 2014) by the American Academy of Sleep Medicine (AASM) [12,73].

**Table 3:** Nosological history of insufficient sleep syndrome as a sleep disorder.

Source	Category
International Classification of Diseases (ICD)	
ICD-8, 1965	780. Certain symptoms referable to nervous system and special senses
	780.6 Disturbance of sleep
ICD-9, 1975	307.4 Specific disorders of sleep of nonorganic origin
	307.41 Transient disorder of initiating or maintaining sleep
	307.42 Persistent disorder of initiating or maintaining sleep
	307.49 Other
ICD-10, 1990 ICD-10-CM, 2018	F51 Nonorganic sleep disorders
	F51.0 Nonorganic insomnia
	F51.8 Other nonorganic sleep disorders
	G47 Sleep disorders
	G47.0 Disorders of initiating and maintaining sleep (insomnias)
	G47.1 Disorders of excessive somnolence (hypersomnias)
ICD-11, 2021	07 Sleep-wake disorders: Hypersomnolence disorders
	7A26 Insufficient sleep syndrome
Diagnostic and Statistical Manual of Mental Disorders (DSM)	
DSM-II, 1968	VII. Special symptoms (306)
	306 Special symptoms not elsewhere classified
	306.4 Disorders of sleep
DSM-III, 1980	A. DIMS: Disorders of initiating and maintaining sleep (insomnias)
	1. Psychophysiological, b. Persistent
	B. Disorders of excessive somnolence (DOES)
	9. Other DOES conditions. b. Insufficient sleep
DSM-IV, 1994	Primary sleep disorders
	Dyssomnia: Primary insomnia, Primary hypersomnia
DSM-5, 2013	307.44 (F51.11) Hypersomnolence disorder
	No specific code for insufficient sleep syndrome
Diagnostic Classification of Sleep and Arousal Disorders (DCSAD)	
DCSAD, 1979	B. Disorders of excessive somnolence (DOES)
	9. Associated with other DOES conditions
	b. Insufficient sleep…. acute sleep deprivation
	C code B.9.b, ICD-9-CM code 307.49-4
International Classification of Sleep Disorders (ICSD)	
ICSD-1, 1990	1. Mental disorders
	a. Developmental sleep disorders
	2. Inadequate sleep hygiene ……. ICD-9-CM code 307.41-1
	3. Insufficient sleep syndrome …. ICD-9-CM code 307.49-4
ICSD-R, 1997	1. Dysomnias
	A. Intrinsic sleep disorders
	1. Psychophysiologic insomnia ICD-9-CM code 307.42-0
	B. Extrinsic sleep disorders
	1. Inadequate sleep hygiene……. ICD-9-CM code 307.41-1
	5. Insufficient sleep syndrome……. ICD-9-CM code 307.49-4
ICSD-2, 2005	A. Insomnia
	5. Inadequate sleep hygiene ICD-10 code F51.0
	C. Hypersomnia of central origin
	7. Behaviorally Induced Insufficient sleep syndrome ICD-10 code F51.8
ICSD-3, 2014	A. Insomnia
	1. Chronic insomnia disorder ICD-9-CM code 307.42, ICSD-10-CM code F51.01
	C. Central disorders of hypersomnolence
	8. Insufficient sleep syndrome ICD-9-CM code: 307.44, ICD-I0-CM code: F51.12

Behaviorally induced CSD was designated as “behaviorally induced insufficient sleep” in the DCSAD and in the first ICSD edition (ICSD-1, 1990) and as “behaviorally induced insufficient sleep syndrome” in the revised edition (ICSD, 1997). In the second edition of ICSD (ICSD-2, 2005), the nosological status of behaviorally induced CSD was elevated from a “sleep disorder not due to known substance or physiologic condition” to a sleep disorder with full-fledged diagnostic criteria under “Central hypersomnia.” ISS cannot be classified as a hypersomnolence disorder in the latest DSM edition (DSM-5) because one of the diagnostic criteria is “self-reported excessive sleepiness (hypersomnolence) despite the main sleep period lasting at least 7 hours” and ISS is, by definition, characterized by a shortened main sleep period [71]. The latest ICSD edition (ICSD-3, 2014) readapted the term “insufficient sleep syndrome” (ISS) for behaviorally induced CSD. ISS is considered a disorder of “central hypersomnolence” with diagnostic codes ICD-9-CM 307.44 and ICD-10-CM F51.12 [12]. A distinction is made in the ICSD-3 between primary disorders of central hypersomnolence (narcolepsy type 1, narcolepsy type 2, idiopathic hypersomnia, and Kline-Levin syndrome) and secondary disorders of central hypersomnolence (hypersomnia due to a medical or psychiatric disorder, hypersomnia due to a drug or substance, and ISS). In the ICSD-3, the term “chronic insomnia disorder” is used for all subtypes of chronic insomnia and inadequate sleep hygiene is no longer required to diagnose any sleep disorder, including insomnia and ISS. The latest ICD (ICD-11, 2021) considers ISS as a diagnostic entity (7A26) under “07 Sleep-wake disorders: Hypersomnolence disorders.”

ISS Diagnostic Criteria and Challenges

The ICSD diagnostic criteria for ISS (ICSD-3, 2014) require the presence of six conditions (Table 4) [12]. ISS is diagnosed in a person who develops CSD due to behaviorally induced (voluntary) habitual (most nights) chronic (≥3 months) curtailment of sleep (daily sleep time < minimum required for age). Sleep duration may be curtailed by delaying its onset and by waking up before enough sleep is obtained, often with the aid of an alarm clock or another person. CSD is often characterized by longer sleep duration during non-working days. Opportunities to sleep ad libitum for many days (e.g., vacations) will reduce or abolish the symptoms of CSD. Even though ISS is “voluntary” it is not necessarily “intentional”. Diagnosing ISS requires detailed information about the person’s past and present sleep routines, including the amount of sleep desired, the amount achievable, and the amount obtained. Sources of information include personal or collateral history, sleep logs, and/or actigraphy.

**Table 4:** ICSD-3 diagnostic criteria for insufficient sleep syndrome. ^1 ^If there is doubt about the accuracy of personal history or sleep logs, then actigraphy should be performed, preferably for at least two weeks. ^2 ^In the case of long sleepers, reported habitual sleep periods may be normal based on age. However, these sleep periods may be insufficient for these patients. Adapted with permission from American Academy of Sleep Medicine [12].

Criteria A to F must be fulfilled.
A. The patient has daily periods of irrepressible need to sleep or daytime lapses into sleep or, in the case of prepubertal children, there is a complaint of behavioral abnormalities attributable to sleepiness.
B. The patient's sleep time, established by personal or collateral history, sleep logs, or actigraphy,^1^ is usually shorter than expected for age.^2^
C. The curtailed sleep pattern is present most days for at least three months.
D. The patient curtails sleep time by such measures as an alarm clock or being awakened by another person and generally sleeps longer when such measures are not used, such as on weekends or vacations.
E. Extension of total sleep time results in resolution of the symptoms of sleepiness.
F. The symptoms are not better explained by another untreated sleep disorder, the effects of medications or drugs, or other medical, neurologic, or mental disorder.

**Figure 1:**
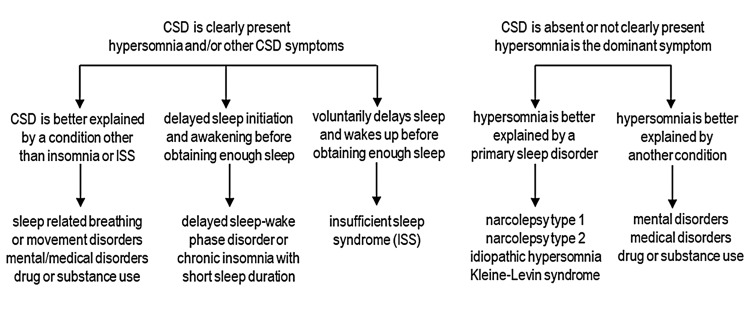
Differential diagnosis of chronic sleep deficiency (CSD) and hypersomnia. Original diagram created by the authors.

ISS must be distinguished from other causes of CSD and from other conditions that cause hypersomnolence (Figure 1). Conditions that can potentially cause CSD include mental, neurologic, or medical disorders, drugs or substances, SBD, sleep-related movement disorders, chronic insomnia with short sleep duration, and circadian sleep disorders, notably delayed sleep-wake phase disorder with short sleep duration. Delayed sleep-wake phase disorder is characterized by a significant delay in the phase of the major sleep episode in relation to the desired sleep time, presumably because of abnormal interaction between the endogenous circadian rhythm and the homeostatic processes that regulate sleep and wakefulness. Insomnia is characterized by persistent difficulty in initiating or maintaining sleep, often attributed to autonomic dysregulation and a state of hyperarousal. By contrast, the ability to initiate and maintain sleep is not impaired in ISS. When given the opportunity to sleep (e.g., during the weekend), a person with delayed sleep-wake phase disorder or insomnia disorder will have difficulty initiating sleep at the desired time; however, a person with ISS will easily fall asleep. Conditions that manifest primarily as hypersomnolence, but are not usually associated with CSD, must also be included in the differential diagnosis of ISS. The first prerequisite to diagnose ISS (daily episodes of hypersomnolence or the behavioral equivalent of hypersomnolence in children) is also a basic feature of narcolepsy. Moreover, the multiple sleep latency test criteria for narcolepsy of at least two sleep-onset REM (rapid eye movement) periods can also be observed in ISS.

Despite the availability of specific diagnostic criteria for ISS and other causes of CSD, many people with ISS remain undiagnosed and untreated. They continue to be sleepy during the day and underperform at work or in school without realizing it. Instead of being concerned about hypersomnolence, some patients with ISS may focus on other CSD symptoms, such as irritability, distractibility, reduced motivation, dysphoria, fatigue, malaise, loss of appetite, incoordination, and muscle pain. This can result in misdiagnosis or misinterpretation of the symptoms as the cause of the sleep disorder. Finally, the diagnosis of ISS is also challenging because insomnia and other causes of CSD may coexist or another sleep disorder, usually delayed sleep-wake phase disorder, may develop and confuse the clinical picture.

Predisposing and Causal Factors

The habitual delay in the major sleep period of individuals with ISS can be attributed to cognitive factors (lack of awareness of the dangers of CSD) or motivational factors (delaying sleep because of social pressure or pleasure) [60,62]. Indeed, a combination of cognitive and motivational factors may be the basis of ISS for most people, with each factor contributing variably to the pathogenesis of CSD from person to person. Healthcare workers and the public are well aware of the concept of inadequate sleep hygiene and its association with insomnia or CSD. Inadequate sleep hygiene must be viewed as a facilitating factor, not the root cause of insomnia or ISS. Indeed, insufficient sleep hygiene is not a prerequisite for diagnosing insomnia or ISS. In a person with CSD, ISS, and inadequate sleep hygiene, a physician (especially one who is not aware of ISS as a diagnostic entity) might come to the wrong conclusion that inadequate sleep hygiene is the only reason for the person’s CSD.

Responding to the challenge

Thanks to public health programs and campaigns: our vision of healthy sleep has become increasingly clear [74]. An important aspect of these initiatives is population-level sleep health surveillance [75]. Toward this end, the US Behavioral Risk Factor Surveillance System (BRFSS) included sleep-related questions since 1995 (During the past 30 days, for about how many days have you felt you did not get enough rest or sleep?) and included sleep questions in the core questions asked on alternating years [76]. However, a later study examining the validity of these sleep questions concluded that only two of the five sleep questions in the BRFSS were reliable in determining sleep quality [77]. Recently, the US National Sleep Foundation developed and validated a Sleep Health Index for assessing sleep health in epidemiological studies [78]. Sleep-related questions have also been integrated in other nationwide surveillance systems, such as the National Health and Nutrition Examination Survey (NHANES), the National Ambulatory Medical Care Survey (NAMCS), the National Hospital Discharge Survey (NHDS), the National Health Interview Survey (NHIS), and the Youth Risk Behavior Survey (YRBS). These surveys did not only assess various aspects of sleep; they also examined the effectiveness of strategies to improve sleep. Unfortunately, these surveys did not distinguish between ISS and insomnia with short sleep duration.

Awareness of healthy sleep has been the focus of several nationwide and global health campaigns. In 2013, the US Centers for Disease Control and Prevention, the American Association of Sleep Medicine, and the Sleep Research Society launched the National Healthy Sleep Awareness Project. This was followed, a year later, by the “Sleep Well, Be Well” campaign. Sleep is a vital component of the Healthy People 2030 initiative which aims to reduce the rate of drowsy driving and increase the proportion of children and adults who get sufficient sleep. At the global level, the World Sleep Society was formed in 2015 to serve as a bridge between sleep societies and cultures for advancing sleep health worldwide [79]. The World Health Organization underscored the importance of healthy sleep to mental well-being and to the workplace [80]. Sleep health and the risks of inadequate sleep have been the topic of many worldwide infographics and online videos.

Not only did the above-mentioned initiatives enhance public awareness of insomnia and OSA; but they also encouraged healthcare professionals to screen for these sleep disorders in their clinics and public health programs. On the other hand, ISS is not yet perceived as a major sleep disorder and as a leading cause of CSD by healthcare workers and the public. ISS continues to be a blind spot in our vision of healthy sleep.

Population-Oriented Approach

Public health and advocacy programs have successfully raised public awareness of CSD but have fallen short in confronting the issue of ISS. CSD can be addressed more effectively if the government and non-government programs emphasize ISS and its behavioral underpinnings. The first step is to recognize ISS as a leading cause of CSD in society and to grant ISS the same priority status that insomnia, OSA, and other causes of CSD have received over the years. Any educational and regulatory efforts targeting CSD must put ISS at the forefront. The behavioral nature of ISS makes it a challenging problem akin to substance abuse and yet it can be amenable to educational and advocacy efforts [74]. The lack of a sense of urgency in promoting CSD/ISS as a risk factor for chronic illnesses, such as obesity, diabetes, hypertension, and cardiovascular disease, may be explained by the delayed emergence of these diseases and by the complex bidirectional relationship between these diseases and CSD/ISS [64].

Cultural beliefs and practices about sleep should be taken into account when designing and implementing programs for promoting healthy sleep. Beliefs and attitudes concerning sleep, the amount of sleep required, and the proper timing of sleep vary among different segments of society and among different countries [63,81]. Cultural and perceptual differences toward sleep may explain the poor sleep quality of some racial/ethnic populations. Changes in sleep-related beliefs and practices with globalization and the spread of technology are not always for the better [27]. Modernization brought about extended working hours, shift work, and abandonment of otherwise culturally appropriate naps [62]. It is only by understanding the beliefs and practices of the target population that we can design effective educational programs for enhancing CSD/ISS awareness.

Academic institutions should be a prime target of public health efforts to promote healthy sleep. The vulnerability of school-age children, college students, and young adults to ISS is enough motivation to add coursework on sleep, CSD, and ISS to the school curriculum. Students must be educated on the detrimental effects of ISS on physical and mental health and school performance [27]. The workplace is another strategic target of public health campaigns and programs that aim to promote healthy sleep. Employers should promote the importance of sleep health to their employees [66]. They should implement snooze-friendly policies and provide napping facilities for their employees, discourage the extended use of electronic devices, and allow for some flexibility in their employees’ working hours [66].

Local governments can promote healthy sleep through policy change [82]. The huge economic impact of CSD should encourage governments to implement policies and enact laws that have a positive impact on sleep health. High-risk age groups and high-risk occupations should be prioritized. For example, avoiding early class start times is advantageous to middle and high school students. Transportation, healthcare, and law enforcement professionals can benefit from properly regulated work hours and schedules. Eliminating daylight-saving time is one strategy for promoting sleep health in the US [74,82]. 

Clinic-Based Approach

Patients with sleep issues, either as a primary complaint or as a comorbidity of other medical disorders, are usually evaluated and treated first by primary care providers, not by sleep physicians. The rarity with which ISS is coded during medical encounters suggests that most primary care providers, and perhaps some sleep specialists, are failing to recognize ISS or are incorrectly diagnosing another sleep disorder (e.g., insomnia) instead of ISS. The ramifications of ISS to health and social functioning should motivate healthcare professionals to adapt effective and efficient methods for recognizing, diagnosing, and managing ISS.

CSD/ISS screening should be performed routinely in the primary care setting. Identifying risk factors for diseases in the subclinical or early stage is cost-effective and is an important aspect of preventive medicine. Patients are routinely screened for risk factors, such as smoking, substance use, depression, and sexual practices but are usually not interrogated about sleep practices and sleep disorders [74]. CSD/ISS screening can be accomplished using a questionnaire, which the patient can work on at home before their clinic appointment or while they are waiting to be seen in the clinic. The screening tool should include questions related to ICSD-3 diagnostic criteria for ISS, among other things.

Risk stratification is cost-effective for diseases that are prevalent but preferentially affect people with a certain demographic profile. It may be prudent to limit ISS screening to patients at high risk for ISS. Although incomplete, some data are available on the risk of ISS in different age groups and occupations [58,59]. For example, middle school, high school, and college students, healthcare workers, and people who work continuously for more than 8 hours a day are at risk for ISS [62]. People with an occupational risk of physical injury during lapses in attention should be screened routinely for CSD/ISS and other sleep disorders.

The bidirectional relationship between CSD/ISS and other medical disorders must be emphasized by healthcare professionals [48]. CSD/ISS interact in complex ways with other medical and psychiatric disorders. Comorbid sleep disorders can impact the treatment and progression of the primary illness. A major source of confusion in patients with CSD/ISS is insomnia, especially insomnia due to inadequate sleep hygiene, and other causes of hypersomnolence, such as SBD/OSA. Many patients may also have ISS as a comorbid condition of other sleep disorders. 

It is important to address the beliefs and attitudes of the individual to improve their sleep quality. Patients should be asked directly what delays their sleep onset, e.g., engagement in social media, playing games, interacting with peers, etc. and what motivates them to delay their sleep. The importance of sleep to overall health should be stressed. Cognitive behavioral therapy, the most effective long-term treatment for insomnia, may also be effective for ISS [83]. Patients should be advised on matters related to sleep hygiene, such as establishing a consistent wake-up time, limiting the time spent in bed on activities other than sleeping (e.g. watching TV, and working), restricting the use of electronic devices before bedtime, and avoiding consumption of substances that impair sleep quality (e.g., caffeinated or alcoholic beverages, nicotine, energy drinks) before bedtime [84].

Unresolved Issues and Future Directions

Current diagnostic tools for assessing sleep deficiency and its effects on health and function are limited by the lack of objectivity, specificity, or ease of use of these tools in the clinical setting. Polysomnography can measure sleep duration and architecture in the sleep laboratory, but this test is not practical or relevant for long-term recording, which is needed to diagnose CSD/ISS. A sleep diary or actigraphy is used to track a person’s sleep-wake pattern over a period of days or weeks. However, sleep diary data are subjective and wrist motion detection with actigraphy only gives a rough estimate of the person’s sleep-wake pattern. Only subjective methods are available for assessing a person’s sleep quality and degree of sleepiness. The multiple sleep latency test measures sleep propensity, the maintenance of wakefulness test assesses the ability to stay awake, and the psychomotor vigilance test detects the level of vigilance during a task. Although objective, these tests are performed by trained professionals in a controlled environment and are therefore not used for long-term recording.

Confounding variables can affect the assessment of CSD and its adverse effects. People with unrecognized CSD may have residual sleep deficits that can potentially alter the degree and type of functional decline from an episode of acute sleep deprivation/restriction. Recovery sleep or naps that alleviate sleepiness or fatigue may not be enough to correct performance deficits [85]. Catch-up sleep, such as weekend sleep extension, may not prevent the long-term negative health effects of CSD [86]. The roles of microsleep and other forms of sleep loss compensation and the efficacy of prophylactic sleep or sleep banking in mitigating the impacts of sleep deprivation/restriction need further investigation [87,88]. Although specific protocols are available for assessing vigilance, cognition, and performance, such functional domains may overlap. It is not always clear if a symptom reflects CSD or another medical condition; e.g. tiredness can mean sleepiness, fatigue, or both [89]. Finally, the cumulative health burden of CSD may be hard to distinguish from the effects of other chronic diseases; the latter can either be the cause or the effect of CSD [48].

Interindividual differences in vulnerability to sleep loss makes it more difficult to assess the immediate or long-term effects of recurrent sleep deprivation/CSD. Neurobehavioral impairment from chronic sleep restriction is trait-like in that highly replicable phenotypic differences exists between individuals [47]. Resilience to sleep loss is task-dependent; both circadian and homeostatic processes contribute to interindividual variability, and dopamine and adenosine have been implicated in the phenotypic differences. Differential vulnerability or resilience to sleep loss may also be related to genetic and epigenetic factors. Furthermore, the risk of developing chronic diseases from CSD may vary from one individual to the other [90]. Such individual susceptibility to the adverse effects of CSD underscores the need for research to identify susceptibility biomarkers. 

Biomarkers and biosensor technology hold the key to a deeper understanding of sleep, sleep deficiency, and sleepiness. Several behavioral and molecular markers of sleep homeostasis and sleepiness have been proposed [91-93]. The research path is still wide open for the identification of clinically relevant sleep biomarkers. This notably includes the “-omics” approach, i.e., transcriptomics, epigenomics, proteomics, and metabolomics. The understanding and availability of risk determinants will allow the development of individualized sleep and performance prediction models. There is an urgent need to enhance and validate biosensors and wearable technologies to determine which devices can be used to accurately track sleep and circadian cycles [94].

## Conclusions

Our awareness of the essential nature of sleep and the risks of CSD to our health has steadily increased over the last three decades. In contrast, our awareness of ISS as a major cause of CSD has been slow to rise. This is alarming since, of the various causes of CSD, ISS appears to have the fastest rate of increase. Indeed, ISS is already the leading cause of CSD in some parts of the world. Acknowledging ISS as a public health priority is a necessary first step in our response to the broad challenge of CSD and CSD-related health consequences. The next step is to confront ISS directly by employing population-oriented and clinic-based strategies.

Public health programs must grant ISS the same level of importance that insomnia, OSA, and other causes of CSD have received over the years. The behavioral underpinnings of ISS make it a challenging problem, akin to substance abuse, and yet it may be amenable to educational and advocacy efforts. Academic institutions should be a prime target of public health efforts to promote healthy sleep. Coursework on sleep and ISS should be part of the curriculum and students should be educated on the detrimental effects of ISS on physical health, mental well-being, and school performance. The workplace should also be a target of public health campaigns and programs that aim to promote healthy sleep. The huge economic impact of CSD should encourage governments to implement policies and enact laws that have a positive impact on sleep health.

Healthcare workers should know the difference between ISS and other causes of CSD, particularly insomnia and delayed sleep-wake phase disorder. The rarity with which ISS is coded during medical encounters suggests that many healthcare providers are failing to recognize ISS or incorrectly diagnosing another sleep disorder (e.g., insomnia) instead of ISS. Clinic patients should be routinely interrogated about their sleep practices and sleep problems. They should be asked directly what delays their sleep onset and what motivates them to sleep at a particular time. CSD/ISS screening can be accomplished using a questionnaire, which the patient can work on at home before their clinic appointment or while they are waiting to be seen in the clinic. Cognitive behavioral therapy, the most effective long-term treatment for insomnia, may also be effective for patients with ISS. However, some modification in the treatment protocol may be necessary because of the fundamental difference in the underlying mechanisms of ISS (psychological and motivational factors) and insomnia (hyperarousal and sympathetic hyperactivity).
